# TRPV4 channels are essential for alveolar epithelial barrier function as protection from lung edema

**DOI:** 10.1172/jci.insight.134464

**Published:** 2020-10-15

**Authors:** Jonas Weber, Suhasini Rajan, Christian Schremmer, Yu-Kai Chao, Gabriela Krasteva-Christ, Martina Kannler, Ali Önder Yildirim, Monika Brosien, Johann Schredelseker, Norbert Weissmann, Christian Grimm, Thomas Gudermann, Alexander Dietrich

**Affiliations:** 1 Walther Straub Institute of Pharmacology and Toxicology, a member of the German Center for Lung Research (DZL), Ludwig Maximilian University of Munich, Munich Germany.; 2 Institute of Anatomy and Cell Biology, School of Medicine, Saarland University, Homburg, Germany.; 3 Comprehensive Pneumology Center, Institute of Lung Biology and Disease, a member of the DZL, Helmholtz Center Munich, German Research Center for Environmental Health, Munich, Germany.; 4 Justus Liebig University Giessen, Cardio-Pulmonary Institute, University of Giessen and Marburg Lung Center, a member of the DZL, Giessen, Germany.

**Keywords:** Cell Biology, Pulmonology, Calcium channels, Ion channels, Pharmacology

## Abstract

Ischemia/reperfusion-induced edema (IRE), one of the most significant causes of mortality after lung transplantation, can be mimicked ex vivo in isolated perfused mouse lungs (IPL). Transient receptor potential vanilloid 4 (TRPV4) is a nonselective cation channel studied in endothelium; however, its role in the lung epithelium remains elusive. Here, we show enhanced IRE in TRPV4-deficient (TRPV4^–/–^) IPL compared with that of WT controls, indicating a protective role of TRPV4 in maintenance of the alveolar epithelial barrier. By immunohistochemistry, mRNA profiling, and electrophysiological characterization, we detected TRPV4 in bronchial epithelium, alveolar epithelial type I (ATI), and alveolar epithelial type II (ATII) cells. Genetic ablation of TRPV4 resulted in reduced expression of the water-conducting aquaporin-5 (AQP-5) channel in ATI cells. Migration of TRPV4^–/–^ ATI cells was reduced, and cell barrier function was impaired. Analysis of isolated primary TRPV4^–/–^ ATII cells revealed a reduced expression of surfactant protein C, and the TRPV4 activator GSK1016790A induced increases in current densities only in WT ATII cells. Moreover, TRPV4^–/–^ lungs of adult mice developed significantly larger mean chord lengths and altered lung function compared with WT lungs. Therefore, our data illustrate essential functions of TRPV4 channels in alveolar epithelial cells and in protection from edema formation.

## Abstract 2

TRPV4, a non-selective cation channel, is essential for alveolar epithelial function and protects from ischemia-reperfusion-induced lung edema.

## Graphical abstract

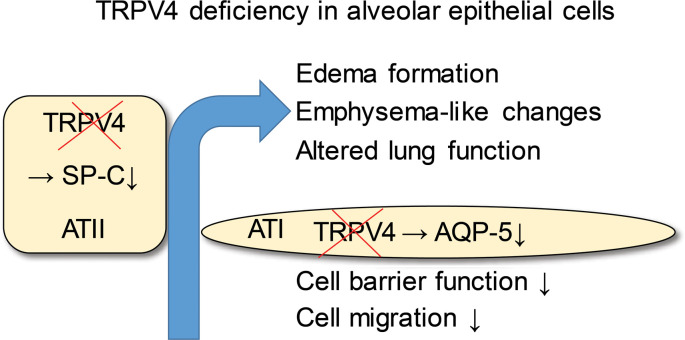

## Introduction

The alveolar epithelium has multiple functions in the lung. On the one hand, the epithelial layer forms a natural barrier to the external environment, protecting the body from invading microorganisms and toxicants, while, on the other hand, alveolar epithelial cells facilitate gas exchange. In the adult lung, the alveolar epithelium consists of 2 epithelial cell types that are crucial to maintain lung homeostasis and tissue repair (1). Alveolar epithelial type I (ATI) cells are elongated with a large surface area and high barrier function, which facilitates gas exchange in close proximity to endothelial cells of the alveolar capillaries (1). ATI cells are also highly water permeable, allowing for ion transport and maintenance of lung fluid balance (2). Although the latter cells cover the largest surface area of the lung (3), alveolar epithelial type II (ATII) cells, which exhibit a cubic morphology, by far outnumber ATI cells (4). ATII cells are also involved in ion transport and liquid homeostasis (5) and are — most importantly — responsible for the production, storage, secretion, and recycling of pulmonary surfactant. Surfactant lowers the surface tension at the tissue-air barrier to allow proper inflation and deflation of the alveoli during breathing (6). Moreover, ATII cells also serve as progenitors for ATI cells and are capable of long-term self-renewal (7). Although alveolar epithelial cells express a wide variety of ion transporters and channels (8), the exact roles of these proteins for specialized alveolar cell functions have remained elusive.

Transient receptor potential vanilloid 4 (TRPV4) is the fourth cloned member of the vanilloid family of TRP channels (9). Like most TRP channels, TRPV4 harbors an invariant sequence, the TRP box (containing the amino acid sequence EWKFAR), in its intracellular C-terminal tail as well as ankyrin repeats in the intracellular N-terminus. The protein is composed of 6 membrane-spanning helices (S1–S6) and a presumed pore-forming loop between S5 and S6 (9, 10). Four of these monomers of the same type preferentially assemble in a functional homotetrameric complex (11), although TRPV4/TRPP2 complexes were also identified in cilia of renal epithelial cells (12). Homotetrameric TRPV4 was originally characterized as a sensor of extracellular osmolarity (13, 14). The channel is functionally expressed in endothelial (15, 16) and epithelial cells of the respiratory system (17–19). TRPV4 channels are thermosensitive in the range of 24°C–38°C and may additionally serve as mechanosensors, because they are activated by membrane and shear stretch as well as by viscous loading (20). As TRPV4 is also involved in pulmonary hypertension (21, 22) and bladder function (23), the channel is an interesting pharmacological target, with numerous modulators already identified (reviewed in ref. 24). Moreover, TRPV4^–/–^ mice were protected from bleomycin-induced pulmonary fibrosis, due to the channel’s constitutive expression and function in lung fibroblasts (25). In lung endothelium, where its role was most extensively studied, direct or indirect activation of TRPV4 by mechanical stress (26), high peak inspiratory pressure (27, 28), and high pulmonary venous pressure due to heart failure (29) resulted in the disruption of the endothelial barrier and edema formation. In other tissues, however, the channel maintains physiological cell barrier, for example, in skin (30), the urogenital tract (31), and the corneal epithelium (32). In tracheal epithelial cells, TRPV4 channels regulate ciliary beat frequency (17), and in alveolar epithelial cells, TRPV4 activation by 4α-phorbol esters produced blebs and breaks in lung septa (33) by unknown molecular mechanisms. Moreover, stimulation of TRPV4 by bacterial LPS mounted a protective response (34), whereas TRPV4 inhibition reduced lung edema and inflammation after chlorine exposure (35). Therefore, TRPV4 channels may function as chemosensors of toxicants in the lung epithelium (reviewed in ref. 36), but their exact role in the alveolar epithelium is still elusive.

We have shown that TRPC6, a member of the classical TRP channel family in the endothelium, increases endothelial permeability during ischemia/reperfusion-induced (I/R-induced) edema formation (37), one of the most significant causes of mortality after lung transplantation. However, as outlined above, endothelial permeability is also increased by TRPV4 activation (summarized in ref. 38). Along these lines, we analyzed I/R-induced edema formation in a TRPV4-deficient (TRPV4^–/–^) mouse model. Surprisingly, edema development was increased in TRPV4^–/–^ lungs, but edema development in TRPC6/TRPV4 double-deficient lungs was similar to that of WT lungs. These data indicate a protective role for TRPV4 channels in the other natural cell barrier of the lung, the epithelium. Therefore, we set out to study functions of TRPV4 channels in the alveolar epithelium, capitalizing on the TRPV4^–/–^ mouse model. Enhanced lung edema formation triggered by I/R probably may be due to downregulation of aquaporin-5 (AQP-5) channels in ATI cells, reduced surfactant protein-C (SP-C) production in ATII cells, and/or emphysema-like changes in the overall lung architecture. Our data suggest an essential role of TRPV4 channels in the alveolar epithelium.

## Results

### Ablation of TRPV4 increases IR-induced edema formation in isolated perfused mouse lungs.

**Figure 1:**
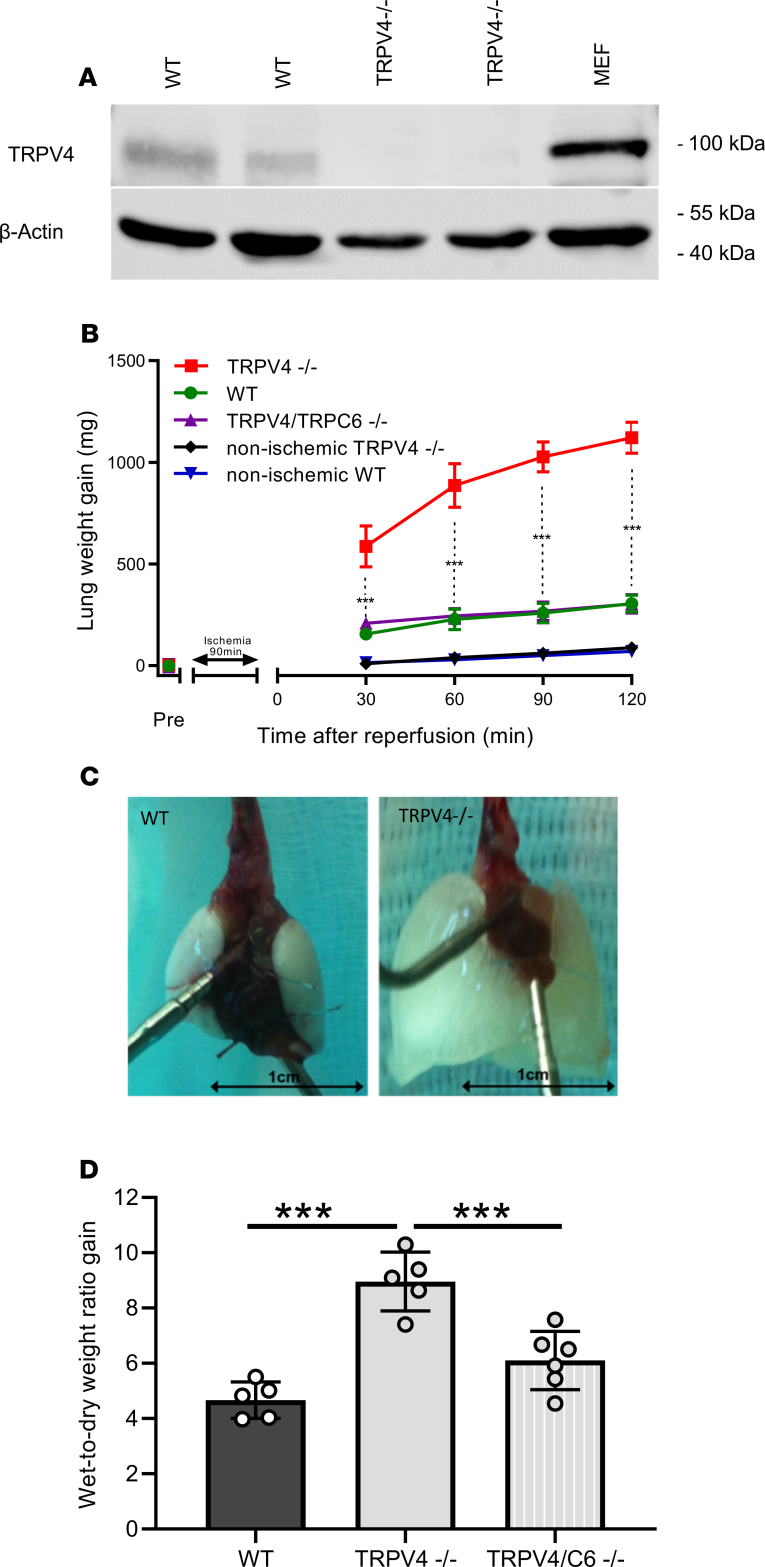
Ablation of TRPV4 increases ischemia-induced edema formation in mouse lungs. (**A**) TRPV4 protein expression in mouse lungs was evaluated by immunoblotting in whole-lung lysates of WT and TRPV4-deficient (TRPV4^−/−^) mice using a TRPV4-specific antiserum. Murine embryonic fibroblasts (MEFs) served as an additional positive control. β-Actin was used as loading control. (**B**) Constant weight measurement of ischemic and nonischemic WT and TRPV4^–/–^ and TRPV4/TRPC6 double-deficient (TRPV4/TRPC6^–/–^) isolated perfused lungs. (**C**) Representative images of WT and TRPV4^–/–^ lungs after ischemia. (**D**) Wet-to-dry weight ratio gains of TRPV4^–/–^ and TRPV4/TRPC6^–/–^ lungs compared with those of WT controls. Data represent mean ± SEM of at least 5 lungs for each genotype. Significance between means was analyzed using ANOVA (**B** and **D**); ***P < 0.001.

To investigate the role of TRPV4 in IR-induced edema formation, we isolated lungs from WT and TRPV4^–/–^ mice. Initial characterization of these mice revealed impaired pressure sensation in dorsal root ganglia (39) and osmotic sensation by exaggerated arginine vasopressin secretion in the brain (40). Loss of TRPV4 protein was confirmed in lung lysates. While in WT controls a protein of appropriately 100 kDa in size was detected by Western blotting with TRPV4-specific antibodies, TRPV4^–/–^ lungs did not express any TRPV4 protein (Figure 1A). Murine embryonic fibroblasts (41), such as pulmonary fibroblasts, express TRPV4 protein (25) and served as an additional positive control. After initial perfusion of isolated lungs for 15 minutes, ischemia was induced for 90 minutes followed by 120 minutes reperfusion. TRPV4^–/–^ lungs showed enhanced lung edema formation, as evidenced by a considerable gain in lung weight, as opposed to WT lungs (Figure 1B), which increased in weight to a similar extent as already described by us previously (37). These results clearly contrast with observations on TRPC6-deficient lungs, which are protected from IR-induced edema due to reduced endothelial permeability (37). Therefore, we generated a TRPV4/TRPC6 double-deficient mouse model (TRPV4/TRPC6^–/–^), which has lungs that lack the increase in IR-induced edema formation but that developed edema, similar to WT mice (Figure 1B). Moreover, lung edema formation in TRPV4^–/–^ lungs was clearly visible by the naked eye (Figure 1C), and, consistently, the wet-to-dry weight ratio increase doubled in TRPV4^–/–^ lungs but only slightly increased in TRPV4/TRPC6^–/–^ lungs (Figure 1D). In conclusion, TRPV4 ablation induces increased IR-induced edema, which can be reduced by additional ablation of TRPC6 channels. To identify a possible role for endothelial TRPV4 channels, which might be activated by shear stress due to hydrostatic pressure (42), we decreased initial flow rates (preflow) from 2 ml to 0.5 ml/min. We did not observe any major changes in edema formation in ischemic and nonischemic WT lungs. TRPV4^–/–^ lungs showed a significantly decreased edema formation only after 90 and 120 minutes of reperfusion for unknown reasons (see Supplemental Figure 1; supplemental material available online with this article; https://doi.org/10.1172/jci.insight.134464DS1).

### TRPV4 is expressed in ATI and ATII cells.

**Figure 2:**
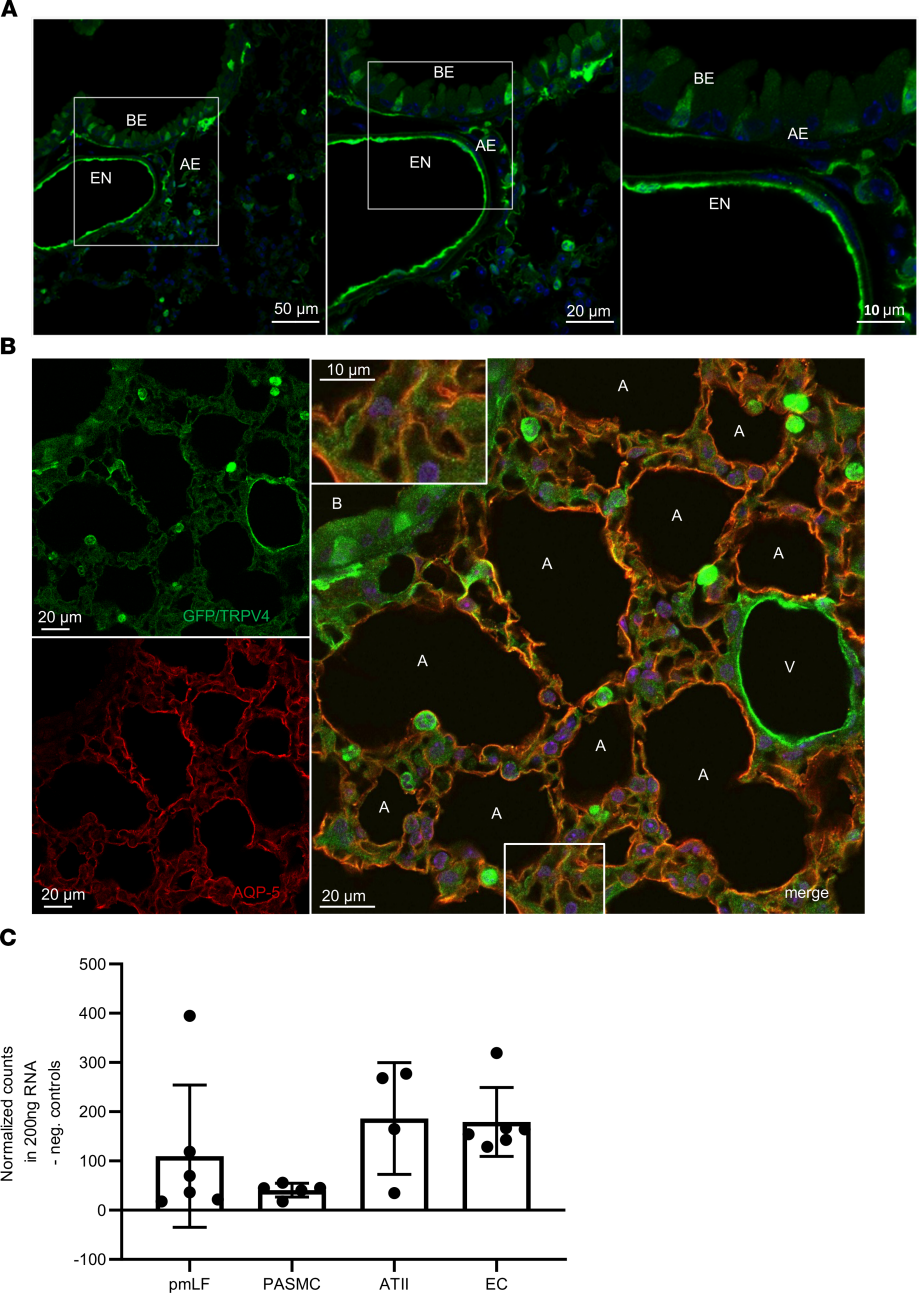
TRPV4 and aquaporin-5 expression in mouse lungs. (**A**) GFP staining (green) by fluorescence-coupled GFP-specific antibodies in lung cryosections of TRPV4EGFP reporter mice reveals expression of TRPV4 in cells of the lung endothelium (EN) as well as in the bronchial (BE) and alveolar epithelium (AE). Nuclei staining was performed with Hoechst dye (blue). Scale bar: 10 μm (right); 20 μm (middle); 50 μm (left). (**B**) Lung cryosections from TRPV4EGFP^–^ reporter mice were stained with fluorescence-coupled antisera directed against GFP and aquaporin-5 (AQP-5). Confocal images were obtained after excitation at 488 nm (for EGFP, left top, green) or after excitation at 561 nm (for AQP-5, left bottom, red). Both images were merged (right). Nuclei staining was performed with Hoechst dye (blue). A, alveolus; B, bronchus; V, vasculature. The inset shows the bottom boxed region in at higher magnification. Scale bar: 10 μm (inset); 20 μm. (**C**) TRPV4 mRNA quantification in lung cells using NanoString technology. ATII, alveolar type II cells; EC, endothelial cells; PASMC, precapillary arterial smooth muscle cells; pmLF, primary murine lung fibroblasts. Data represent mean ± SEM from at least 3 independent cell isolations.

As TRPV4 is highly expressed in lung endothelium, and its activation results in an increase of endothelial permeability (reviewed in ref. 38), we focused on its possible functions in the epithelium. Epithelial cells represent the second natural barrier regulating edema formation. Analysis of mice carrying an EGFP reporter protein under the control of the TRPV4 promoter/enhancer region revealed expression of TRPV4 protein in endothelium as well as bronchial and alveolar epithelium (Figure 2A). In the bronchial epithelium we detected TRPV4 in ciliated cells by costaining with a β-tubulin IV antibody (Supplemental Figure 2, A–C). Neither club nor neuroendocrine cells showed TRPV4 expression (Supplemental Figure 2, D–I). In the alveoli, costaining experiments with an antibody directed against AQP-5 (Figure 2B), a marker protein of ATI cells, which are involved in lung septa formation (2), revealed a red staining indicative of AQP-5 expression in the plasma membrane and an additional green staining of the cytosol, reflecting TRPV4 expression in these cells (Figure 2B, inset). Moreover, direct quantification of TRPV4 mRNA revealed similar expression levels in ATII cells as in lung endothelial cells, but lower mRNA expression in pulmonary murine lung fibroblasts and precapillary arterial smooth muscle cells (Figure 2C). Therefore, TRPV4 channels are expressed in ATI and ATII cells of the alveolar epithelium.

### Loss of TRPV4 resulted in decreased AQP-5 expression in ATI cells.

**Figure 3:**
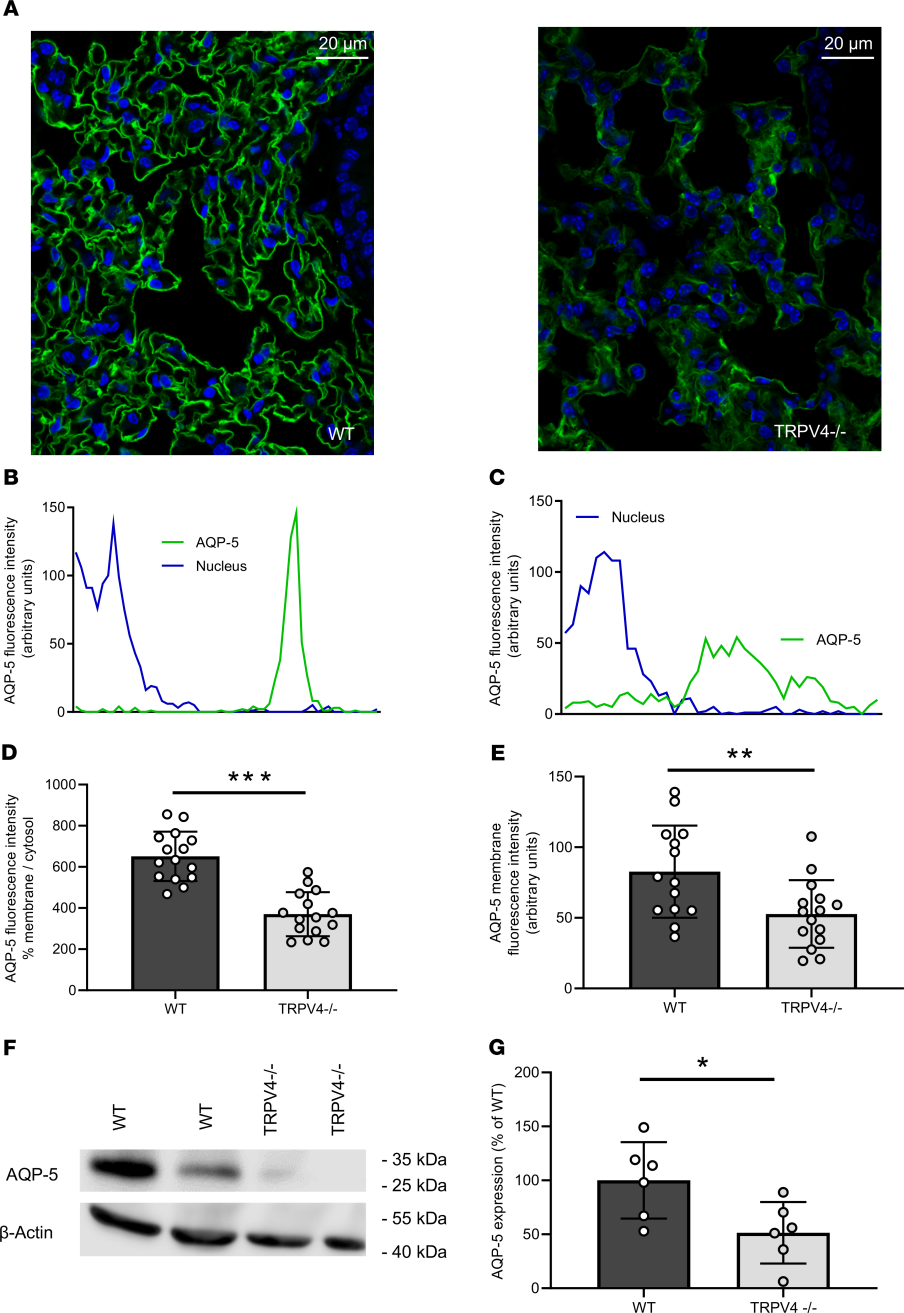
Aquaporin-5 expression and translocation to the plasma membrane in WT and TRPV4^–/–^ alveolar epithelial type I cells. (**A**) Cryosections of WT and TRPV4^–/–^ lungs stained with an aquaporin-5–specific (AQP-5–specific) fluorescence-coupled antibody. Nuclei staining was performed with Hoechst dye (blue). Scale bar: 20 μm. Representative histograms for the quantification of AQP-5 protein in the plasma membrane of WT (**B**) and TRPV4-deficient alveolar epithelial type I (ATI) cells (**C**). (**D** and **E**) Summaries of AQP-5 protein expression in plasma membranes (**D**, percentage of aquaporin-5 membrane/cytosol; **E**, percentage AQP-5 in membranes). Representative Western blot analysis of AQP-5 expression in WT and TRPV4^–/–^ whole-lung lysates (**F**) and summary of AQP-5 expression in lung lysates of TRPV4^–/–^ and WT mice (**G**). Data represent mean ± SEM from at least 6 lungs for each genotype. Significance between means was analyzed using 2-tailed unpaired Student’s *t* test; *P < 0.05, **P < 0.01, ***P < 0.001.

Staining of lung slices with fluorescence-coupled antibodies specific for the water-conducting channel AQP-5 revealed lower total expression levels in ATI cells and reduced plasma membrane localization in TRPV4^–/–^ lungs compared with that in WT lungs (Figure 3, A–E). These results were confirmed by Western blotting of lung lysates probed with an AQP-5–specific antibody (Figure 3, F and G). In clear contrast to these results, protein levels of AQP-1, a major aquaporin channel in the microvascular endothelium, were not significantly different in TRPV4^–/–^ cells compared with WT endothelial cells (Supplemental Figure 3, A–E). Therefore, AQP-5 protein levels in the alveolar epithelium, but not AQP-1 expression in the endothelium is reduced by ablation of TRPV4.

### Identification of currents induced by the TRPV4 activator GSK1016790A only in primary ATII cells from WT mice.

**Figure 4:**
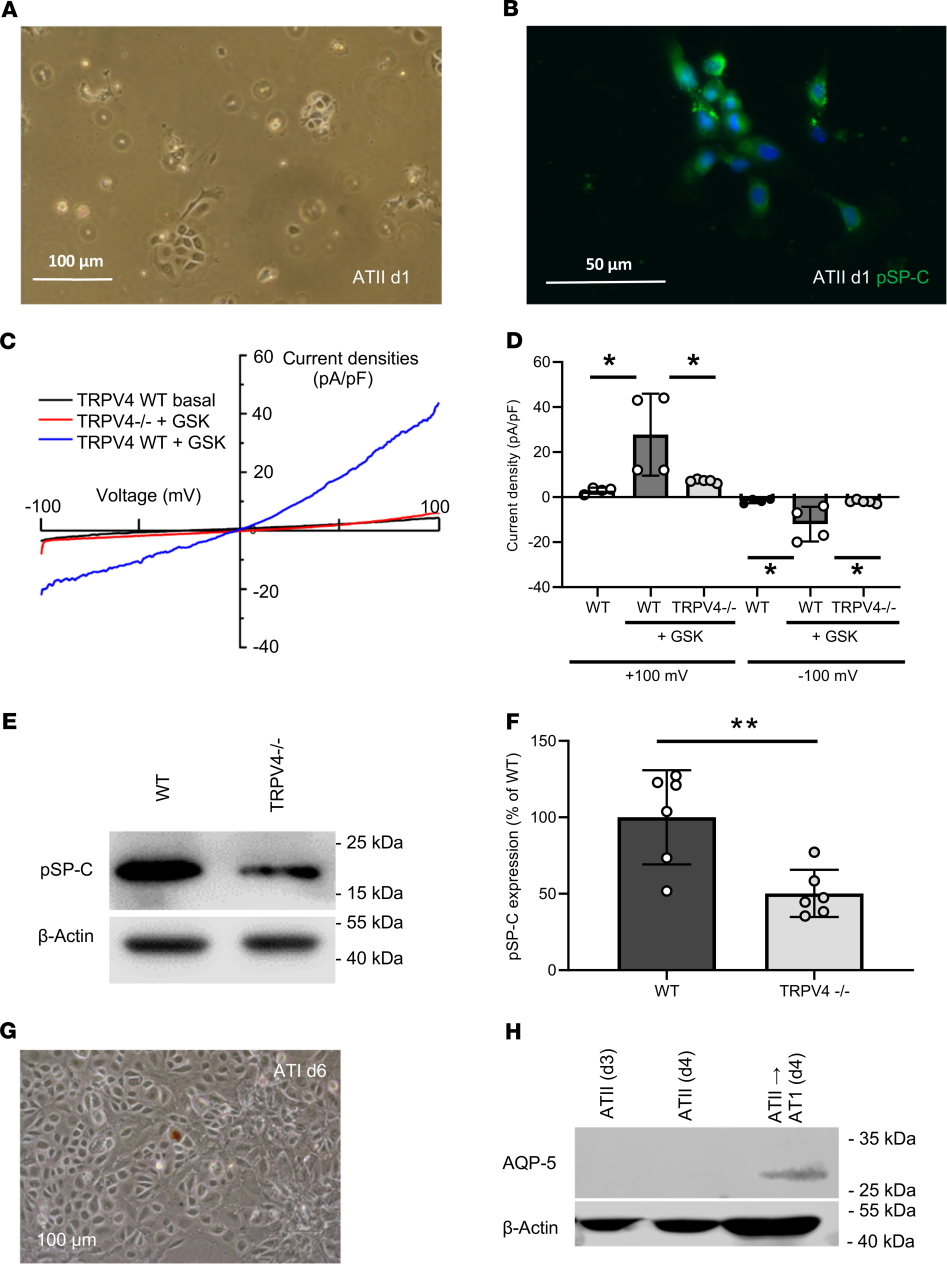
Identification of alveolar epithelial type II cells and differentiation to alveolar epithelial type I cells. Representative cell cluster 1 day after isolation in a phase-contrast image (scale bar: 100 μm) (**A**) and stained with a fluorescence-coupled specific pro-surfactant protein-C (pSP-C) antibody (scale bar: 50 μm) (**B**). Nuclei staining was performed with Hoechst dye (blue). Electrophysiological whole-cell measurements of basal and GSK-induced (GSK-induced) current densities in WT and TRPV4^–/–^ primary alveolar epithelial type II (ATII) cells (**C** and **D**). Representative current density–voltage curves of WT (gray, blue traces) and TRPV4^–/–^ (red trace) ATII cells before (gray trace) and during application of GSK (blue and red traces) (**C**). Summary of current densities at ±100 mV before (white bars) and after application of GSK analyzed in WT (black bars) and TRPV4^–/–^ (gray bars) ATII cells (**D**). Representative Western blot analysis of pSP-C expression in WT and TRPV4^–/–^ ATII cells (**E**) and summary of pSP-C expression in TRPV4^–/–^ and WT ATII cells (**F**). Image of confluent cells on day 6 after ATII cell isolation (scale bar: 100 μm) (**G**) and analysis of AQP-5 expression in cells grown for 3, 4, and 6 days in plastic cell culture dishes by Western blotting (**H**). β-Actin was used as loading control in each blot. Data represent mean ± SEM from at least 3 independent cell preparations of 5 mice each. Significance between means was analyzed using 1-way ANOVA (**C**) or 2-tailed unpaired Student’s *t* test (**F**); *P < 0.05, **P < 0.01.

To investigate the role of TRPV4 on at a cellular level, we first isolated ATII epithelial cells (Figure 4A) from WT and TRPV4^–/–^ mice. We were not able to detect any morphological differences in ATII cells of the different genotypes by phase-contrast microscopy. ATII cells were identified by staining with fluorophore-coupled antibodies directed against directed against prosurfactant protein C (pSP-C) (Figure 4B), which is secreted by ATII cells (reviewed in ref. 5). Patch clamp analysis of primary ATII cells revealed significantly larger currents, which were induced by the selective TRPV4 activator GSK1016790A (GSK, reviewed in ref. 24) only in WT cells, while currents after the application of GSK in TRPV4^–/–^ cells were not significantly different compared with basal currents in WT cells (Figure 4, C and D). Western blotting of protein lysates from ATII cells revealed lower pSP-C levels in TRPV4^–/–^ ATII cells compared with WT cells (Figure 4, E and F). We then differentiated ATII cells to ATI cells by growing them to confluence in plastic cell culture dishes for at least 6 days as described previously (1) (Figure 4G). After 6 days, WT cells expressed AQP-5 protein as an ATI cell marker (Figure 4H). In conclusion, TRPV4 channels are functionally active in ATII cells and are involved in the expression of pSP-C of these alveolar epithelial cells, which can be differentiated to ATI cells in vitro.

### TRPV4^–/–^ ATI cells express less AQP-5, show reduced nuclear localization of NFAT, and decreased cell migration and adhesion.

**Figure 5:**
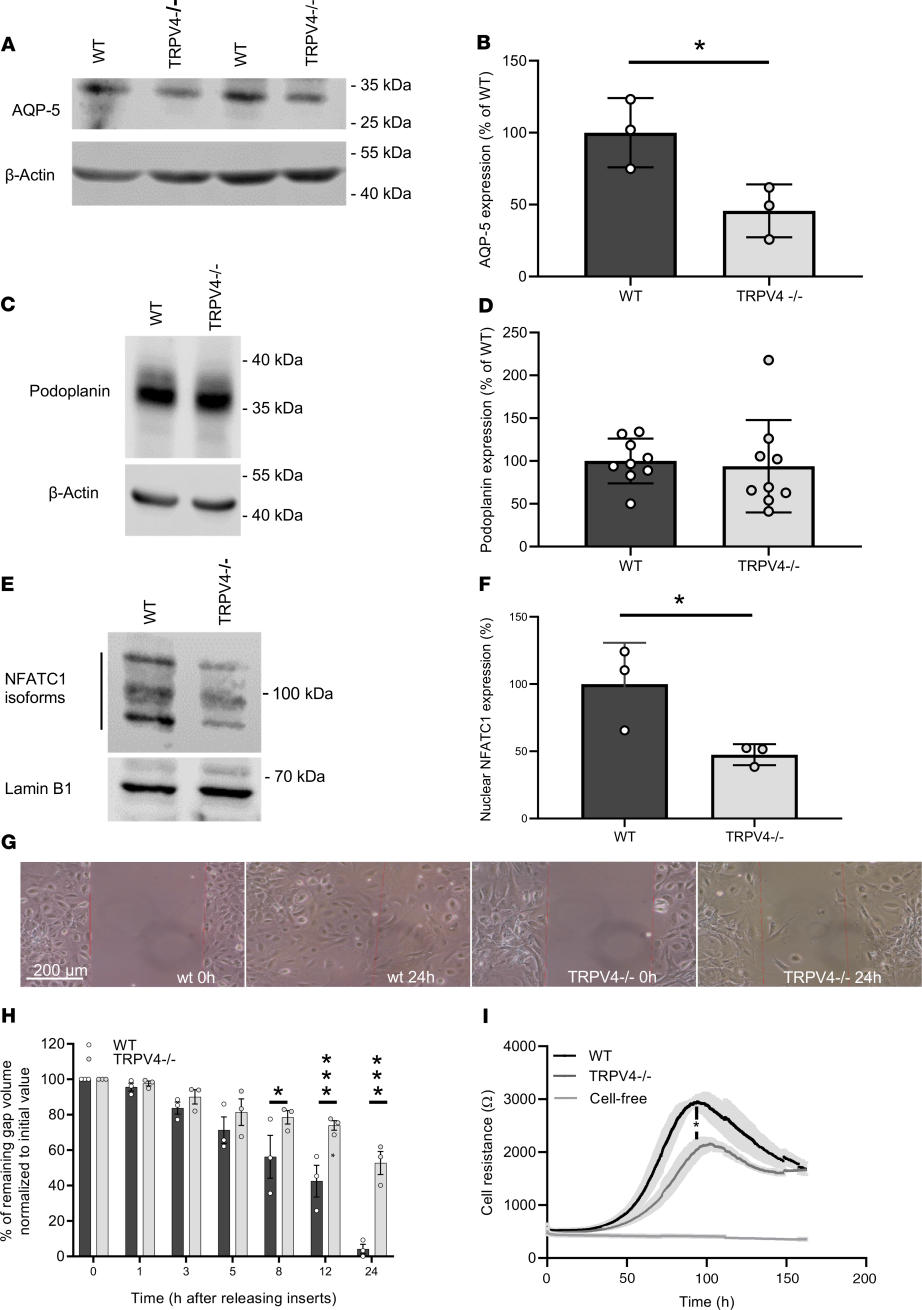
Nuclear localization of nuclear factor of activated T cells in and migration and adhesion of TRPV4-deficient and WT ATI cells. Representative Western blot analysis of AQP-5 expression in WT and TRPV4^–/–^ ATII cells differentiated to ATI cells (**A**) and summary of AQP-5 expression in these cells (**B**). Representative Western blot analysis of podoplanin expression — another ATI cell marker — in WT and TRPV4^–/–^ ATII cells differentiated to ATI cells (**C**) and summary of podoplanin expression in these cells (**D**). Representative Western blot analysis of nuclear NFATc1 localization in WT and TRPV4^–/–^ ATI cells (**E**) and summary of nuclear factor of activated T cells (NFAT) localization in these cells (**F**). Lamin B1 served as loading control. Representative images of a migration assay after removing inserts (scale bar: 200 μm) (**G**). Summary of remaining gap values normalized to initial values quantified in migration assays of TRPV4^–/–^ and WT ATI cells after releasing inserts at 0, 1, 3, 5, 8, 12, and 24 hours (**H**). Electrical cell resistance was quantified with an ECIS device for WT and TRPV4^–/–^ ATI cells for 160 hours. (**I**). Data represent mean ± SEM from at least 3 independent cell preparations of 5 mice each. Significance between means was analyzed using 2-tailed unpaired Student’s *t* test; *P < 0.05, ***P < 0.001.

As already shown in lung sections of TRPV4^–/–^ mice, translocation of AQP-5 to the plasma membrane was reduced in TRPV4^–/–^ cells (Figure 5, A and B). To test if TRPV4^–/–^ ATII cells are able to differentiate to ATI cells, we analyzed the expression of podoplanin (T1α), another ATI cell marker protein. Notably, podoplanin expression was not significantly different in TRPV4^–/–^ ATII cells differentiated to ATI cells (Figure 5, C and D).To further analyze ATI cell function, we quantified nuclear NFATc1 levels. The translocation of NFATc1 protein to the nucleus was significantly reduced in TRPV4^–/–^ cells (Figure 5, E and F). Moreover, cell migration analyzed by gap closure in in vitro experiments was clearly slowed down in TRPV4^–/–^ ATI cells compared with WT cells (Figure 5, G and H). As an additional line of evidence, we transfected ATII cells with TRPV4-specific or control siRNAs, differentiated them to ATI cells, and quantified cell migration in the same way (Supplemental Figure 4). Most interestingly, we obtained similar results in cells transfected with TRPV4 siRNA, which showed a significantly slower migration compared with nontransfected cells as well as cells transfected with the control siRNAs. As determined by electrical cell impedance sensing (ECIS), subconfluent TRPV4^–/–^ ATI cells showed reduced cell barrier function (Figure 5I). Therefore, ablation of TRPV4 induced less AQP-5 expression, reduced nuclear localization of nuclear factor of activated T cells (NFAT), and reduced cell migration and cell barrier function.

### TRPV4^–/–^ mice showed emphysema-like lung structure and altered lung function.

**Figure 6:**
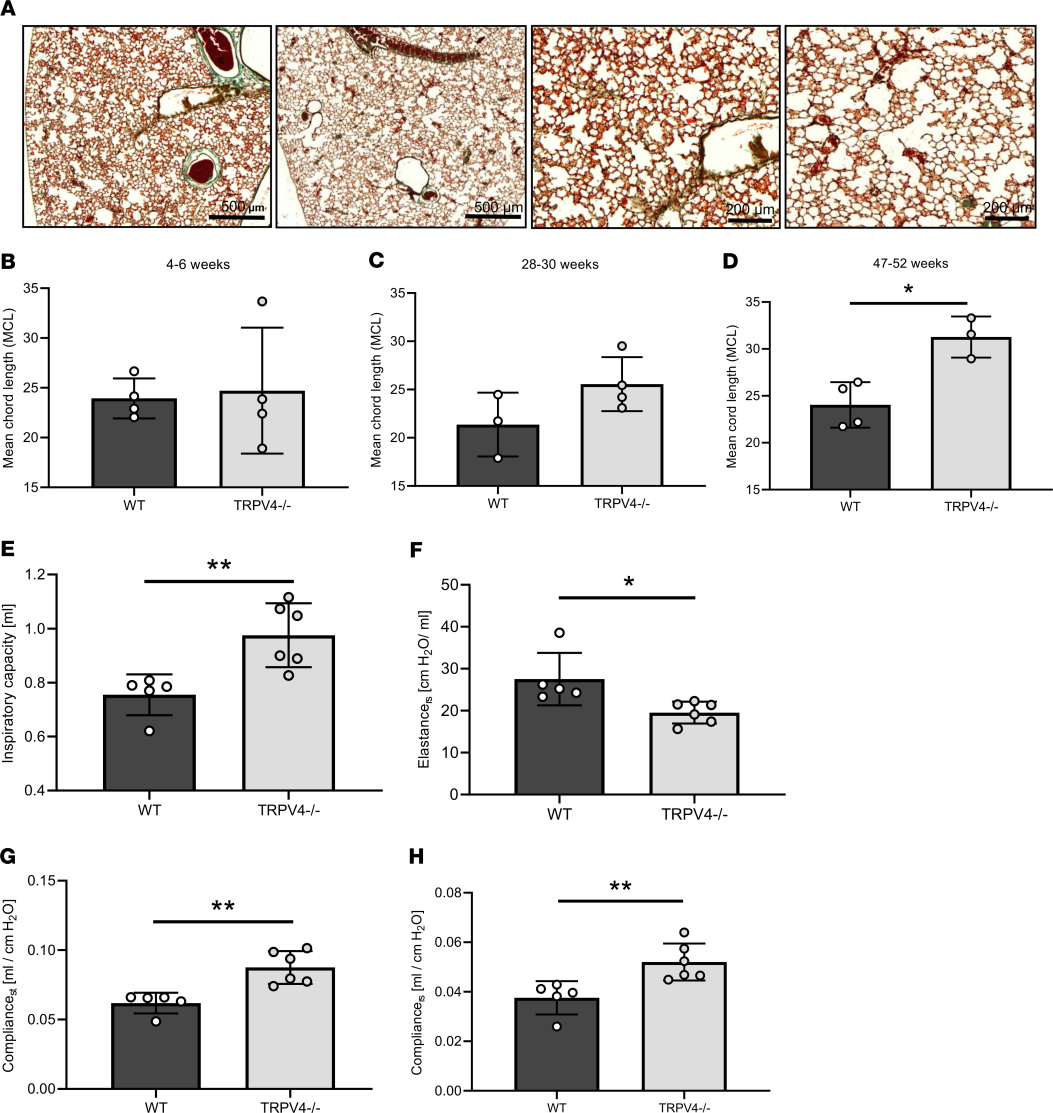
Chord lengths and lung function of WT and TRPV4^–/–^ mice. Representative images of Masson’s trichrome–stained lung sections from 52-week-old WT (left, scale bar: 500 μm) and TRPV4^–/–^ mice (right, scale bar: 200 μm) (**A**). Quantification of mean chord lengths of 4- to 6- (**B**), 28- to 30- (**C**), and 47- to 52-week-old (**D**) WT and TRPV4^–/–^ mice. Inspiratory capacity (**E**), elastance of the respiratory system (**F**), static compliance (C_st_, **G**) and compliance of the respiratory system (C_rs_, **H**) of 6-month-old WT and TRPV4^–/–^ mice. Data represent mean ± SEM from at least 3 mice. Significance between means was analyzed using 2-tailed unpaired Student’s *t* test; *P < 0.05, **P < 0.01.

To analyze differences in lung anatomy as a consequence of altered ATI cell function, we quantified mean chord lengths (MCLs) in histological lung sections (Figure 6A). TRPV4 ablation significantly increased MCL of the alveolar lumen in adult (47–52 week old, Figure 6D) mice compared with WT lungs, while young mice (4–6 weeks old) showed no differences (Figure 6B). Lungs from 28- to 30-week-old mice were also prone to larger MCLs (Figure 6C), which, however, were not significantly different compared with those of WT lungs. Moreover, lung function was altered (Figure 6, E–H): TRPV4^–/–^ lungs showed increased inspiratory capacity and compliance (Figure 6, E, G, and H) as well as decreased elastance (Figure 6F), which was significantly different from that of WT mice of the same age. In conclusion, adult TRPV4^–/–^ mice showed emphysema-like changes in their lungs, which may be responsible for altered lung function.

## Discussion

Although TRPV4 is highly expressed in lungs, its exact function is still elusive (reviewed in ref. 24). Activation of TRPV4 in endothelial cells by mechanical stress, for example, stretching (27, 28, 43), as well as oxidative stress by exposure to H_2_O_2_ (44) resulted in an increased Ca^2+^ influx mediated by the channel and an increase in endothelial permeability conducive to lung edema (reviewed in ref. 38). Along these lines, pharmacological blockade of TRPV4, for example, by the specific blocker HC-067047, decreased intracellular Ca^2+^ levels in endothelial cells and protected mice from vascular leakage and lung injury (28). Expression and function of TRPV4 channels in the alveolar epithelium, however, has not been studied yet.

Here, we quantified IR-induced edema as one of the most common and significant causes of morbidity and mortality after lung transplantation (45), using the isolated perfused lung model (37). Much to our surprise, TRPV4^–/–^ lungs were not protected from IR-induced lung edema, as observed in TRPC6^–/–^ mice (37). On the contrary, genetic TRPV4 ablation resulted in a robust increase in lung edema (Figure 1B) and a higher wet-to-dry weight ratio increase (Figure 1D) when compared with that of control WT mice. Barrier function was rescued by consecutive breeding of TRPV4^–/–^ mice with TRPC6^–/–^ mice, because lung edema formation in double-deficient mice was similar to that in WT animals (Figure 1B).

As TRPV4 activation in endothelial cells has been shown to result in higher edema formation, we focused on the lung epithelium, another physiological cell barrier in the lung. Recent publications indicate an epithelial function of the channel opposed to that in endothelium, i.e., stabilization of the epithelial barrier in the skin (30), the urogenital tract (31), and the corneal epithelium (32). We demonstrated TRPV4 expression in ATI and ATII cells (Figure 2, B and C). Our further molecular analysis corroborated a functional link between TRPV4 and AQP-5, a water-conducting channel expressed in ATI cells (46). Hypotonic solutions increased the association and surface localization of TRPV4 and AQP-5 in salivary gland cells (47), and AQP-5 expression is regulated by TRPV4 in lung epithelial cells (48). Most interestingly, the expression and plasma membrane translocation of AQP-5 channels in ATI cells were significantly reduced (Figure 3). Therefore, TRPV4 channels increase AQP-5 expression and translocation in ATI cells in clear contrast to human bronchial epithelial cells, where it has been reported that activation of TRPV4 channels by shear stress decreased AQP-5 levels (47). To analyze TRPV4 function on a cellular level, we isolated ATII cells identified by their expression of pSP-C (Figure 4, A and B). We detected significantly larger currents induced by the TRPV4 activator GSK in WT but not in TRPV4^–/–^ ATII cells (Figure 4, C and D). To our knowledge, these data show for the first time that TRPV4 channels are not only expressed, also functional in ATII cells. Quantifying pSP-C levels by Western blotting revealed a reduced expression in TRPV4^–/–^ cells compared with that in WT cells (Figure 4, E and F). The role of surfactant proteins in the prevention of alveolar edema by reducing surface tension as a driving force for fluid flow across the air-blood barrier is still a matter of debate (49) but might also explain exaggerated edema formation in TRPV4^–/–^ mice. Therefore, functional TRPV4 and TRPC6 channels are not only located in different cell types, such as alveolar epithelial and lung endothelial cells, respectively, but may have different roles by decreasing or increasing IR-induced edema. TRPV4 channels aid in epithelial barrier function by supporting SPC production and reducing edema formation in a chronic manner, while TRPC6 channels acutely increase endothelial permeability during IR-induced edema formation (37). Although we cannot exclude a role for endothelial TRPV4 channels, it is unlikely that TRPV4 channels in the endothelium are activated by shear stress due to hydrostatic pressure, as reducing the preflow in the experiments had no effect on IR-induced edema formation (Supplemental Figure 1).

Next, we differentiated ATII cells to ATI cells (1), monitored by the expression of 2 ATI cell markers: AQP-5 and podoplanin. As AQP-5 protein expression was reduced in TRPV4^–/–^ ATI cells (Figure 5, A and B), while podoplanin levels were not altered (Figure 5, C and D), it seems rather unlikely that TRPV4 deficiency and/or a reduction of pSP-C expression results in reduced ATII cell to ATI cell differentiation in general. Plasma membrane translocation of AQP-5 as well as AQP-5 expression may depend on nuclear localization of the transcription factor NFAT by an increase of intracellular Ca^2+^ via TRPV4 similar to TRPC channels (50). Therefore, we quantified nuclear NFAT levels and detected significantly lower levels in TRPV4^–/–^ cells in comparison with that in WT control cells (Figure 5, E and F). A major breakthrough in our understanding of AQP-5 function for water transport across apical membranes of ATI cells was the analysis of AQP-5–deficient mice (51). Although lack of AQP-5 entailed a 10-fold decrease in alveolar permeability in response to an osmotic gradient, AQP-5^–/–^ mice are indistinguishable from WT mice with regard to hydrostatic pulmonary edema as well as isoosmolar fluid transport from the alveolar space (51, 52). Cognizant of this scenario, a role for AQP-5 in the clearance of fluid from the alveolar space after IR-induced lung edema cannot entirely be ruled out, but it appears to be unlikely, and we tried to dissect other additional mechanisms for the vulnerability of TRPV4^–/–^ lungs to edema formation.

As 2 reports demonstrated decreased migration of human epithelial ovarian cancer (53) or endometrial adenocarcinoma cells (54) after downregulation of AQP-5, we set out to quantify cell migration of ATII cells differentiated to ATI cells. TRPV4^–/–^ ATI cells showed a clear deficit in closing gaps by cell migration after releasing inserts compared with WT cells (Figure 5, G and H). In additional experiments, we were able to reproduce these results in cells transfected with TRPV4 siRNAs compared with nontransfected cells as well as cells transfected with control siRNAs (Supplemental Figure 4). These data suggest an important role of TRPV4 channels in cell migration, which needs to be further analyzed in the future. Moreover, cell resistance, as analyzed by ECIS, was significantly reduced in growing TRPV4^–/–^ ATI cells in contrast to that in WT cells (Figure 5I). Both cell types, however, reached confluence after 160 hours, excluding gross changes in their proliferation rates. Changes in cell morphology were also not detected by microscopy.

ATII cells are able to differentiate to ATI cells after lung injury during repair processes in adult mice (7) to reestablish barrier function of the lung alveolus. Therefore, we analyzed lung alveolar histology in WT and TRPV4^–/–^ lungs in young and adult mice. MCL as a measure of alveolar size was increased in adult (47–52 weeks old) but not in young (3 weeks old) TRPV4^–/–^ mice compared with WT mice of the same ages (Figure 6, A–D). We concluded that differences were not caused by defects in embryonic lung development but were due to ongoing growth and repair processes in adult animals. Most interestingly, the emphysema-like changes in lung morphology were also detected in SP-C–deficient mice (55), raising the possibility that reduced SP-C levels in TRPV4^–/–^ ATII cells may also contribute to the phenotype. In the same vein, adult TRPV4^–/–^ mice showed altered lung function, with increased inspiratory capacity and compliance as well as decreased elastance (Figure 6, E–H) compared with WT mice of the same ages. Loss of septa formation because of reduced SP-C levels in adult TRPV4^–/–^ mice may be responsible for decreased clearance of fluid from the alveolar space and may therefore explain higher levels of edema formation in TRPV4^–/–^ lungs.

In summary, loss of TRPV4 channels in alveolar epithelial cells results in decreased pSP-C production in ATII cells and lower AQP-5 expression and membrane localization in ATI cells. The latter proteins are likely to be involved in continuously ongoing repair processes in adult mice, resulting in emphysema-like changes in TRPV4^–/–^ mice. These chronic events may define a protective function of TRPV4 channels against lung edema formation, in clear contrast to their acute detrimental role in endothelial cells.

## Methods

### Animals.

TRPC6^–/–^ (56) and TRPV4^–/–^ (B6.199X1-Trpv4^tm1MSZ^ from Riken BioResource Center, RBRC01939) (39, 40) mice were backcrossed 10 times to the C57/BL6J strain. TRPC6/TRPV4^–/–^ mice were obtained by crossing both gene-deficient mouse models. TRPV4EGFP reporter mice (Tg(TRPV4-EGFP)MT43Gsat/Mmucd from MMRC) were bred as previously described (57). Sex- and age-matched mice older than 3 months were used in the experiments, if not mentioned otherwise in the figure legends.

### Isolated, perfused mouse lung.

Quantification of edema formation in isolated perfused mouse lungs was done as described previously (37). In brief, mice were anesthetized by intraperitoneal injection of ketamine (100 mg/kg BW), xylazine (0.7 mg/kg BW), and anticoagulated with heparin (500 IU/kg BW). Animals were intubated via a small incision in the trachea, ligated, and ventilated with room air using the VCM type 681 (positive end−expiratory pressure, 3 cmH_2_O; positive end–inspiratory pressure 3 cmH_2_O; respiratory rate was 90 breaths/min). The sternum was opened, the ribs were spread, and the right ventricle was incised to place the air-free perfusion catheter into the pulmonary artery. After ligation, the perfusion was started with 0.5 ml/min perfusion solution (7.19 g sodium chloride, 0.33 g potassium chloride, 0.27 g magnesium hexahydrate, 0.36 g calcium chloride dihydrate, 0.15 g potassium dihydrogen orthophosphate, 2.67 g glucose monohydrate, 51.28 g hydroxyethyl starch [type 2000000/05] ad 1000 mL with aqua ad iniectabilia, and 0.1848 mg/mL sodium hydrogen carbonate to adjust pH to 7.3) using an ISMATEC Tubing Pump. A second perfusion catheter was introduced in the left ventricle and secured by ligation. The lung, the trachea, and the heart were excised from the thorax in 1 piece and transferred to a 37°C temperature-equilibrated housing chamber for the perfused mouse lung model (IPL-2, Hugo Sachs Elektronik/Harvard Apparatus). The perfusion was slowly raised stepwise to 2 ml/min, and perfusion pressure was monitored with the PLUGSYS TAM-A/P75 type 17111 (Harvard Apparatus). Weight changes were constantly measured with the edema Balance Module/EBM type 713 (Harvard Apparatus). Data were monitored with Pulmodyn software (Harvard Apparatus). The perfusion pressure during the measurements was not significantly different between genotypes as well as before and after ischemia.

### Analysis of functional parameters of the respiratory tract.

Mice were anesthetized with ketamine (270 mg/kg BW) and xylazin (11 mg/kg BW), intratracheally intubated through a small incision of the trachea, and connected to the ﬂexiVent system (Scireq).

### Immunohistochemistry.

Mouse lungs were inﬂated with 2.5% (m/v) glutaraldehyde in PBS and processed for paraffin or O.C.T. compound (Tissue-Tek, Sakura Finetek) embedding. Paraffin-embedded tissue sections (3 μm) were cut using a microtome (Zeiss), mounted on glass slides, deparaffinized in xylene, and rehydrated in graded alcohol. Masson Goldner trichrome staining (Masson Goldner Trichrome Staining Kit, Carl Roth 3459) was done according to the manufacturer’s instruction with iron hematoxylin solution for 8 minutes, Goldner’s stain 1 for 6 minutes, Goldner’s stain 2 for 1 minute, and Goldner’s stain 3 for 5 minutes. After dehydration in 100% EtOH and clearing in xylol twice for 1 minute, the sections were mounted in Roti-Histokit II (Carl Roth T160.2). Sections were analyzed by design-based stereology using an Olympus BX51 light microscope equipped with the new Computer Assisted Stereological Toolbox (newCAST, Visiopharm) as described previously (58). For MCL measurements, 10–20 frames were selected randomly across multiple sections by the software, using the ×20 objective, and superimposed by a line grid and points. The intercepts of lines on alveolar wall (Lsepta) and points localized on air space (Pair) were counted and calculated as follows: MCL (∑Pair × L(p)/∑Isepta × 0.5, where L(p) is the line length per point). Cryo-embedded lungs were cut in 10 μm sections on a cryostat (Leica), mounted on glass slides, and surrounded with a hydrophobic pen (Vector Laboratories). After washing with PBS, the sections were blocked for 30 minutes in PBS containing 0.2% Triton X-100 and 5% NGS. Incubation with primary antibody was done at 4°C overnight and secondary antibody at room temperature for 1 hour. Antibodies were diluted in blocking solution. After nuclei staining with Hoechst dye (Thermo Fisher Scientific) (2 μg/mL) for 5 minutes at room temperature followed by sufficient washing the sections were mounted in Roti-Histokit II. The following antibodies and dilutions were used: anti-GFP (chicken, Thermo Fisher Scientific, A10262, 1:200), anti–β-tubulin IV (rabbit monoclonal, Abcam, 179509, 1:1600), anti–AQP-1 (rabbit, Alomone Labs, AQP-001, 1:100), anti–AQP-5 (rabbit, Alomone Labs, AQP-005, 1:100), anti-CC10 (mouse, Santa Cruz Biotechnology, E-11, 1:200), anti-chicken (goat, Thermo Fisher Scientific, A11039, 1:400), anti-CGRP (goat, 1:400, Acris, BP022), anti-rabbit IgG (goat, coupled to Alexa Fluor 488, Thermo Fisher Scientific, A32731, 1:500 and donkey, coupled to Cy3, Merck Millipore, AP182C, 1:1000), and anti-goat IgG (donkey, Life Technologies, A11058, 1:400). For direct labeling of the anti-CC10 antibody, the Zenon Alexa Fluor 546 mouse IgG_1_ kit was used according to the manufacturer’s recommendations (Invitrogen, 25004). Stained cryosections were analyzed on an epifluorescence microscope (Zeiss Imager.M2, Carl Zeiss) and on a confocal microscope (LSM 880, Carl Zeiss). For membrane localization analysis, staining intensity was analyzed along a line from the nucleus into the cytosol and the plasma membrane.

### Primary murine alveolar epithelial cells.

Isolation of ATII cells was done as described previously (1, 59, 60). In brief, lungs were flushed via a catheter through the pulmonary artery with 0.9% NaCl solution (B. Braun Melsungen AG), inflated with 1 mL dispase (BD Biosciences), followed by 500 μl 1% low-melting-point agarose (MilliporeSigma), and incubated for 1 hour at room temperature. Subsequently, lung lobes were separated and dissected using 2 forceps; filtered through 100 μm, 20 μm, and 10 μm nylon filters (Sefar); and centrifuged for 10 minutes at 200*g*. Cell pellets were resuspended in DMEM (MilliporeSigma) and plated on CD45- and CD16/32-coated (BD Biosciences) culture dishes for a negative selection of macrophages and lymphocytes and incubated for 30 minutes at 37°C. Nonadherent cells were collected and seeded on uncoated dishes to negatively select fibroblasts at 37°C for 25 minutes. Cells were collected and identified by staining with a fluorescent coupled anti pSP-C antibody (Chemicon International, AB3786, 1:20000). Live cells were counted by trypan blue staining in a Neubauer counting chamber. 2 × 10^6^ cells/well of a 6-well plate were seeded in DMEM containing 10% FCS (Invitrogen), 1% HEPES (Carl Roth), and 1% penicillin/streptomycin (Lonza), and used for analysis or grown for at least 6 days for ATI cell differentiation. ATII cells were transfected with 1 μM Accell SMARTpool siRNA for TRPV4 (in starving medium, 0.1% FCS) 2 days after isolation. On day 6, the cells were washed once and kept in starving medium. A noncoding pool of the Accell siRNA in starving medium served as control (see Table 1 for siRNA sequences).

### Patch-clamp recordings of ATII cells.

Conventional whole-cell recordings were carried out at room temperature 24 hours after isolation of ATII cells from WT and TRPV4^–/–^ mice. The following bath solution, containing 140 mM NaCl, 1.3 mM MgCl_2_, 2.4 mM CaCl_2_, 10 mM glucose, 10 mM HEPES (pH 7.4 with NaOH) and resulting in an osmolality of 310 mOsm/kg, was used for patch-clamp recordings. The pipette solution contained 135 mM CsCl, 2 mM Na-ATP, 1 mM MgCl_2_, 5 mM EGTA, and 10 mM HEPES (pH 7.2 with CsOH), resulting in an osmolality of 296 mOsm/kg. Patch pipettes made of borosilicate glass (Science Products) had resistances of 2.2–3.5 MΩ for whole-cell measurements. Data were collected with an EPC10 patch clamp amplifier (HEKA) using the Patchmaster software. Current density-voltage relations were obtained before and after application of the TRPV4 activator GSK (1 mM) to the bath solution using voltage ramps from –100 to +100 mV, each lasting 5 seconds. Data were acquired at a frequency of 40 kHz after filtering at 2.8 kHz. The current density-voltage curves and the current density amplitudes at ±100 mV were extracted at minimal or maximal currents, respectively.

### Western blot analysis.

Western blotting was done as previously described (61). Chemiluminescence was detected in an Odyssey Fc unit (Licor). The following antibodies and dilutions were used: HRP-conjugated anti–β-actin antibody (MilliporeSigma, A3854HRP, 1:10000), anti-TRPV4 (rabbit, Abcam, ab 39260, 1:1000), anti–AQP-5 (rabbit, Alomone AQP-005, 1:1000), anti-Podoplanin (goat, R&D Systems, AF3244, 1:500), secondary anti-goat IgG (whole molecule) peroxidase (MilliporeSigma, A5420-1ML, 1:10000), and secondary anti-rabbit IgG peroxidase (POX) antibody (MilliporeSigma, A6154, 1:10000). One representative of three Western blots is shown in the figures.

### NanoString nCounter expression analysis.

Direct quantification of TRPV4 mRNA in murine lung cells was done as described previously (62). In brief, total RNA from pulmonary murine cells was isolated using the Qia RNeasy Mini Kit (QIAGEN). Quantity, purity, and integrity of the RNA samples were controlled by spectrophotometry (NanoQuant). Two probes (the reporter and the capture probe) were hybridized to their specific target mRNAs. Then, the target-probe complexes were immobilized in the imaging surface of the nCounter Cartridge by binding of the capture probe. Finally, the sample cartridges were scanned by an automated fluorescence microscope, and molecular barcodes (fluorophores contained in the reporter probe) for each specific target were counted. For expression analysis by nCounter NanoString technology, 200 ng total RNA was hybridized with a NanoString Gene Expression CodeSet and analyzed using the nCounter Digital Analyzer. Background correction was performed and normalization was applied using 4 different housekeeping genes (succinate dehydrogenase subunit A [Sdha], β2-microglobulin, GAPDH, and β-actin). The DNA sequences used for mRNA expression analysis are summarized in Table 2.

### Migration assay.

Around 4.4 × 10^6^ ATII cells/well were seeded on a 2-well silicone insert with a 500 μm cell-free gap (ibidi GmbH) and grown in DMEM (10% FCS, 1% HEPES, and 1% penicillin/streptomycin) for 5 days to obtain ATI-like cells. Subsequently, cells were starved in serum-reduced medium (0.1% FCS) for 24 hours before insert detachment to create a defined cell-free gap. Images were taken 0, 1, 3, 5, 8, 12, and 24 hours after gap creation. Migration was analyzed by measuring the remaining gap width with ImageJ software (NIH) in 3 images per time point and replicate.

### Isolation of nuclear fractions.

Isolation of nuclear protein extracts from ATI-like cells after 6 days of culture was performed with a Nuclear Extract Kit according to the manufacturer’s instructions (Active Motif, 40010) as described previously (61). In brief, cells were first washed with PBS containing phosphatase inhibitors. Cytoplasmic protein fractions were collected by adding hypotonic lysis buffer and detergent, causing leakage of cytoplasmic proteins into the supernatant. After centrifugation (14,000*g* for 30 seconds), nuclear protein fractions were obtained by resuspending pellets in detergent-free lysis buffer containing protease inhibitors. NFAT proteins were analyzed by Western blotting as described below using an NFATc1-specific (mouse, Santa Cruz Biotechnology, sc-7294, 1:600) antiserum and lamin B1 (rabbit, Thermo Fisher Scientific, PA5-19468, 1:5000) antibodies as loading controls. Protein bands were normalized to loading controls and quantified by an Odyssey Fc unit (Licor).

### Quantification of cell resistance by ECIS.

Resistance changes of ATII cells differentiated to ATI cells were analyzed using an ECIS device (Applied Biophysics). Freshly isolated epithelial cells were seeded on ECIS culture ware (8W10E+; Applied Biophysics), which was preincubated with FCS for 3 hours and connected to the ECIS device. A total of 1 × 10^4^ cells was seeded per chamber and grown at 37°C and 5% CO_2_ in an incubator. Resistance (Ω) was analyzed at 2000 Hz over 160 hours.

### Statistics.

All statistical test were performed using GraphPad Prism 7. Numbers of mice and cells as well as statistical tests used are indicated in the figure legends and include 1-way ANOVA and 2-tailed unpaired Student’s *t* test. A *P* value of less than 0.05 was considered significant.

### Study approval.

All animal experiments were approved by the local authority (Regierung Oberbayern, Munich, Germany).

## Author contributions

JW and AD conceived the study, analyzed data, and wrote the manuscript. MB, NW, AÖY, JS, and CG aided in the experimental design. JW, YKC, CS, MK, GKC, and SR conducted the experiments. TG aided in critical analysis and in revising the manuscript. All authors read the manuscript and provided critical revisions.

## Supplementary Material

supplemental data

## Figures and Tables

**Table 2:**
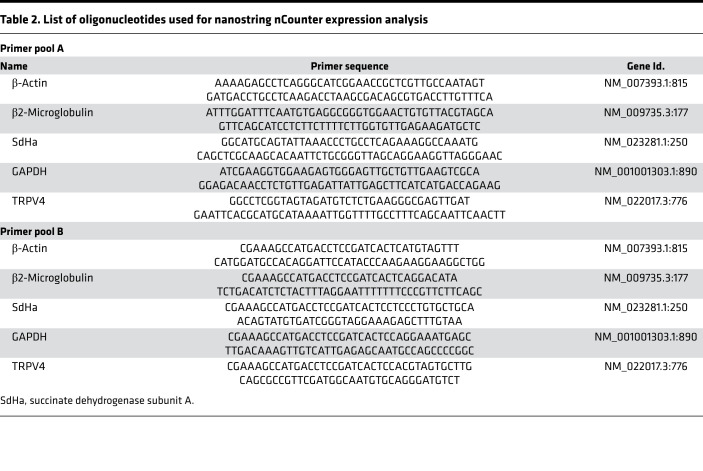
List of oligonucleotides used for nanostring nCounter expression analysis

**Table 1:**
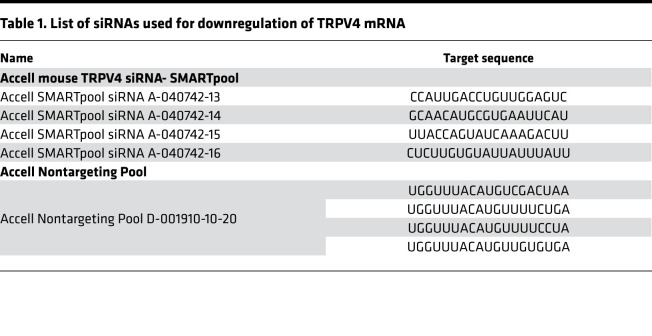
List of siRNAs used for downregulation of TRPV4 mRNA

## Version 1 09/15/2020

In-Press Preview

## Version 2 10/15/2020

Electronic publication
