# Supplementary material for: TRPV4 channels are essential for alveolar epithelial barrier function as protection from lung edema
Source: JCI Insight. 2020 Oct 15;5(20):e134464. doi: 10.1172/jci.insight.134464 (PMC7605532; doi:10.1172/jci.insight.134464)
Supplement: supplemental data [file jciinsight-5-134464-s164.pdf]

Supplemental Figures

TRPV4 channels are essential for alveolar epithelial barrier function as protection from lung edema

Jonas Weber, Suhasini Rajan, Christian Schremmer, Yu-Kai Chao, Gabriela Krasteva-Christ, Martina Kannler, Ali Önder Yildirim, Monika Brosien, Johann Schredelseker, Norbert Weissmann, Christian Grimm, Thomas Gudermann, Alexander Dietrich

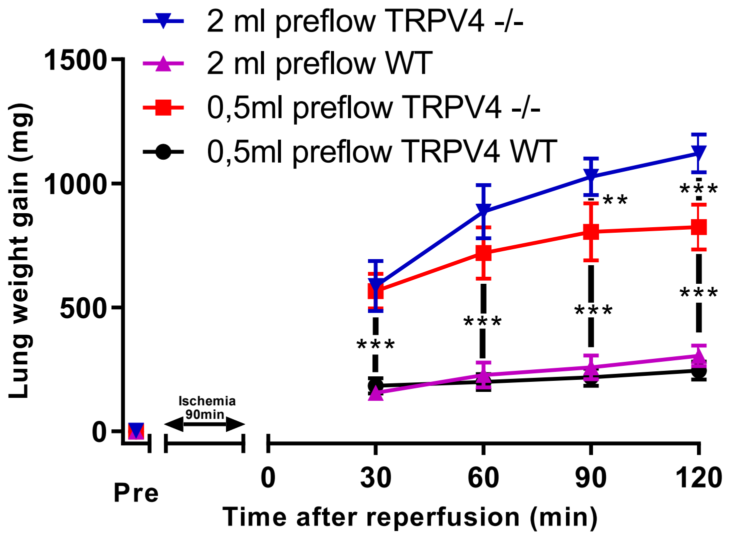

Supplemental Figure 1. Constant weight measurements of ischemic WT and TRPV4-/ isolated perfused lungs after applying preflows (Pre) of different velocities (0.5 ml versus 2ml). Significance between means was analyzed using two way ANOVA and indicated as *** for $p<0.001$ and ** for $p<0.01$.Ablation of TRPV4 increases ischemia-induced edema formation in mouse lungs.

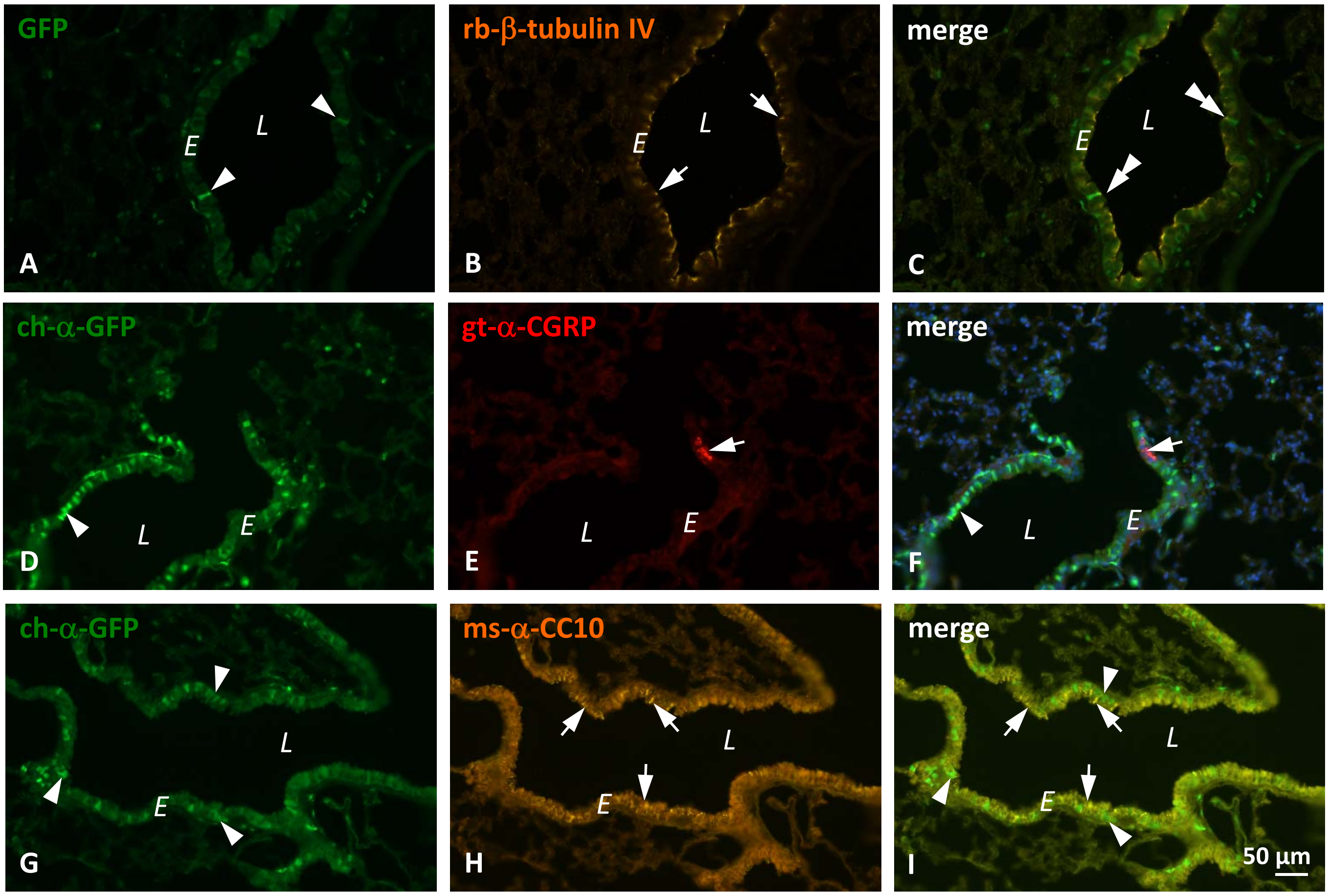

Supplemental Figure 2. Localization of TRPV4 in mouse lungs using immunohistochemistry. *L* = bronchial lumen, *E* = epithelial layer, scale bars = 50 µm. (**A**) Lung cryosections of TRPV4-eGFP reporter mice revealed expression of TRPV4 (arrowheads) in a subpopulation of bronchial epithelial cells. (**B**). Ciliated cells were labeled for β-tubulin IV (arrows). (**C**) A merged view of images shown in *A* and *B*. TRPV4eGFP-positive cells are positive for β tubulin IV (double arrowheads, **A-C**). **D-F**) TRPV4eGFP-fluorescence was enhanced using an anti-GFP-antibody. The same distribution pattern of TRPV4eGFP-immunoreactive cells (arrowheads, **D-F**) was observed. Neuroepithelial bodies labeled by anti-CGRP-antiserum (arrows, E-F) were not immunoreactive for TRPV4eGFP *(merge,* **F***)*. *(***G-I***)* TRPV4eGFP-immunoreactive cells (arrowheads, **G**) were not labeled for CC10 (arrows, **H-I**), a marker for club cells.

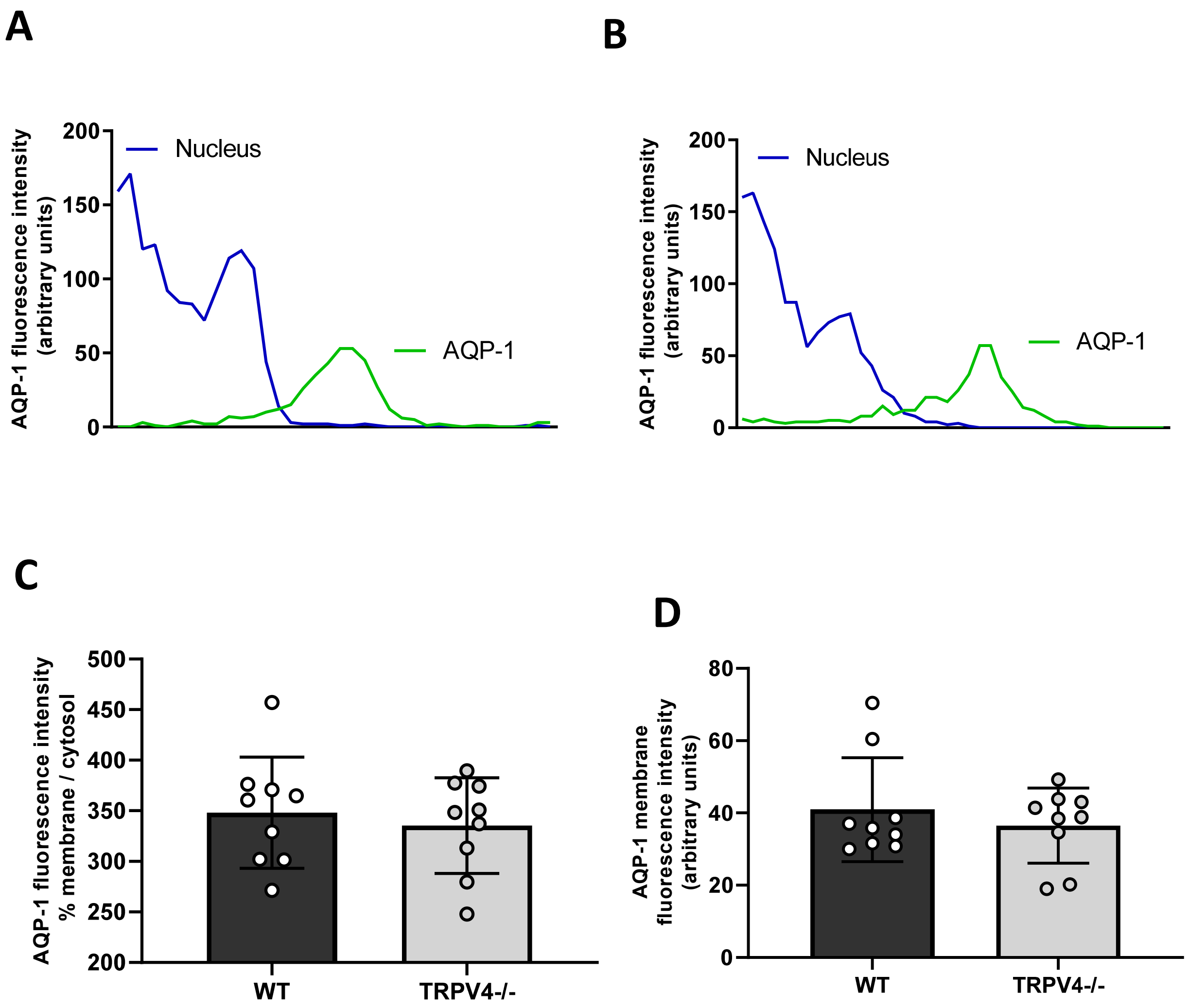

Supplemental Figure 3. Aquaporin-1 (AQP-1) expression and translocation to the plasma membrane in WT and TRPV4-/- endothelial cells. Representative histograms for the quantification of AQP-1 protein in the plasma membrane of WT (**A**) and TRPV4-deficient endothelial cells (**B**). Summaries of AQP-1 protein expression in plasma membranes (% AQP-1 in membranes (**C**)) and in relation to the cytosol (% AQP-1 membrane/cytosol (**D**)). Data represent means ± SEM from 9 lungs. No significance between means was identified using two tailed unpaired Student's t-test.

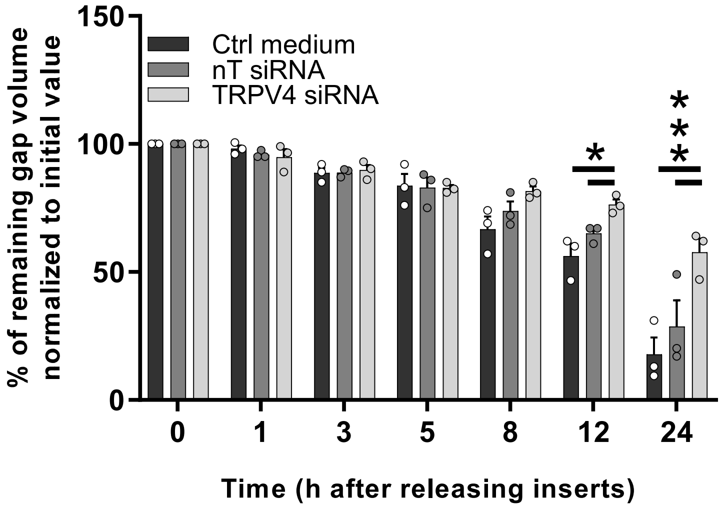

Supplemental Figure 4. Migration of ATI cells expressing TRPV4 siRNA. Summary of remaining gap values normalized to initial values quantified in migration assays of WT ATI cells (control (ctrl) medium) as well as ATI cells transfected with siRNA (non-targeting (nT siRNA) or TRPV4-specific siRNAs (TRPV4 siRNA)) after removing inserts at 0, 1, 3, 5, 8, 12 and 24 h. Data represent means ± SEM from 3 independent cell preparations of 5 mice each. Significance between means was analyzed using two way ANOVA and indicated as *** for $p<0.001$ and * for $p<0.05$.
